# Comparative analysis of nuclear and mitochondrial DNA from tissue and liquid biopsies of colorectal cancer patients

**DOI:** 10.1038/s41598-021-95006-6

**Published:** 2021-08-18

**Authors:** Anna Haupts, Anne Vogel, Sebastian Foersch, Monika Hartmann, Annett Maderer, Nicolas Wachter, Tobias Huber, Werner Kneist, Wilfried Roth, Hauke Lang, Markus Moehler, Nils Hartmann

**Affiliations:** 1 Institute of Pathology, University Medical Center JGU Mainz, Langenbeckstraße 1, 55131 Mainz, Germany; 2 Department of Internal Medicine I, University Medical Center JGU Mainz, Langenbeckstraße 1, 55131 Mainz, Germany; 3 Department of General, Visceral and Transplantation Surgery, University Medical Center JGU Mainz, Langenbeckstraße 1, 55131 Mainz, Germany; 4 Present Address: Department of General and Visceral Surgery, St. Georg Hospital Eisenach gGmbH, Mühlhäuser Straße 94, 99817 Eisenach, Germany

**Keywords:** Cancer genetics, Cancer genomics, Gastrointestinal cancer, Tumour biomarkers, Cancer

## Abstract

The current standard for molecular profiling of colorectal cancer (CRC) is using resected or biopsied tissue specimens. However, they are limited regarding sampling frequency, representation of tumor heterogeneity, and sampling can expose patients to adverse side effects. The analysis of cell-free DNA (cfDNA) from blood plasma, which is part of a liquid biopsy, is minimally invasive and in principle enables detection of all tumor-specific mutations. Here, we analyzed cfDNA originating from nucleus and mitochondria and investigated their characteristics and mutation status in a cohort of 18 CRC patients and 10 healthy controls using targeted next-generation sequencing (NGS) and digital PCR. Longitudinal analyses of nuclear cfDNA level and size during chemotherapy revealed a decreasing cfDNA content and a shift from short to long fragments, indicating an appropriate therapy response, while shortened cfDNAs and increased cfDNA content corresponded with tumor recurrence. Comparative NGS analysis of nuclear tissue and plasma DNA demonstrated a good patient-level concordance and cfDNA revealed additional variants in three of the cases. Analysis of mitochondrial cfDNA surprisingly revealed a higher plasma copy number in healthy subjects than in CRC patients. These results highlight the potential clinical utility of liquid biopsies in routine diagnostics and surveillance of CRC patients as complementation to tissue biopsies or as an attractive alternative in cases where tissue biopsies are risky or the quantity/quality does not allow testing.

## Introduction

Colorectal cancer (CRC) is the third most commonly diagnosed cancer and the fourth most common cause of cancer death worldwide^1,2^. The current standard for characterizing colorectal carcinomas is histopathological examination of the biopsy or resection tissue specimens with subsequent determination of molecular markers in formalin-fixed paraffin-embedded (FFPE) DNA. However, analysis of tumor tissue samples are invasive and do not provide full representation of both spatial and temporal tumor heterogeneity^3^. In contrast, analysis of circulating tumor-derived DNA from blood plasma, which is part of a liquid biopsy, enables minimally invasive detection of in principle all tumor-specific alterations and their dynamic changes. Several studies demonstrated good concordance between profiling molecular alterations in FFPE DNA and matched circulating tumor DNA (ctDNA) from CRC patients, and in addition revealed that plasma analysis could be more informative than corresponding analysis of tissue biopsies^4–7^. The majority of cell-free DNA (cfDNA) is released by apoptotic cells with a modal size of 166 bp, resulting in a “ladder” pattern depending on nuclease action during apoptosis^8,9^. Therefore, cfDNA is highly fragmented and was shown to have an even shorter fragment size in the circulation of cancer patients compared to those derived from non-neoplastic cells^10–14^. Furthermore, quantitative analysis of cfDNA levels have been shown to yield important prognostic value, for example, an increase of concentration correlated with tumor stages and overall survival in CRC patients^6,15^. Hence, analysis of cfDNA level and fragmentation pattern could potentially serve to distinguish between cancer patients and healthy subjects or to follow the course of disease. However, quantitative and qualitative analyses of cfDNA are very challenging and therefore are still rarely used in routine diagnostic of CRC.

Moreover, the proportion of an individual’s tumor-derived ctDNA of total cfDNA is often small (< 1% according to some studies) but tumor fraction can vary between cancer types and even between patients with histologically identical cancers^6,16^. In general, metastatic patients yield a higher amount of tumor-derived plasma DNA but even patients with advanced stage diseases can harbor only a low amount of ctDNA^17,18^. Furthermore, cfDNA is also released by non-neoplastic cells, resulting in a high wildtype background^16,18^. Thus, analysis of the limited number of ctDNA molecules requires highly sensitive molecular approaches. A high level of sensitivity is achieved by PCR-based techniques, such as digital PCR (dPCR), which rely on detection of specific known mutations using primers that are complementary to the mutant sequences^19^. Next-generation sequencing (NGS)-based techniques also have been implemented to simultaneously investigate multiple molecular alterations to enable genome-wide analysis or targeted sequencing of a subset of genes^20^. However, genomic profiling from ctDNA analysis has difficulties and standards for core quality metrics, such as coverage and sensitivity, have to be defined with regard to the interpretation of variants found near to the limit of detection^21^.

Besides cell nuclei, mitochondria have their own circular genome and thus contribute to total cfDNA content in blood. A single cell contains up to several thousand copies of mitochondrial DNA (mtDNA) opposed to two copies of nuclear DNA (nDNA)^22^. Hence, examination of cell-free mitochondrial DNA (cf-mtDNA) theoretically could offer a higher level of sensitivity than analyzing cell-free nuclear DNA (cf-nDNA). In addition, mtDNA has a high mutation rate and in CRC and other cancers underlying molecular alterations have been reported^23–25^. Thus, evaluation of cf-mtDNA as potential biomarker is interesting for liquid biopsies because high copy number could enable detection of even small amounts of ctDNA and their molecular alterations. Furthermore, previous studies have shown that cf-mtDNA content and fragmentation pattern differentiate between cancer patients and healthy individuals, thus also potentially serving as indicative marker of disease^8,26,27^. However, cf-mtDNA has been not fully characterized yet and an efficient approach for comprehensive analysis is still lacking.

Here, we report a proof-of-principle for evaluation of liquid biopsy workflows for potential application in clinical routine of CRC patients. We examined cfDNA from nuclear and mitochondrial origin, involving determination of cfDNA levels and fragment sizes from healthy controls and CRC patients at baseline and also during chemotherapy, as well as analyses of molecular alterations and copy numbers. We validated the analytical sensitivity of the applied molecular approaches (NGS and dPCR) using reference material with known mutations and investigated paired tissue and plasma mutation analysis concerning sequence variants and copy number variations (CNVs), and identified analytical factors that hamper concordance between both.

## Results

### Patient characteristics

The 18 patients included in this study had non-metastatic (n = 9, 50%), locally advanced (n = 3, 16.7%) or metastatic (n = 6, 33.3%) CRC. Table 1 provides an overview of patients’ clinicopathological features. Overall, 33.3% (n = 6) of the primary tumors were localized in the colon (sigmoid colon: n = 3, ascending colon: n = 1, descending colon: n = 1, cecum: n = 1) and 66.7% (n = 12) in the rectum. According to this, 22.2% (4/6) had a left-sided and 11.1% (2/6) had a right-sided primary colon tumor. The most frequent metastatic sites were lymph nodes followed by liver. Almost all patients (17/18) had microsatellite-stable tumors (94.4%). More than half of the patients (11/18) received radiochemotherapy (n = 3, 16.7%) or chemotherapy (n = 8, 44.4%). The pretherapeutical CEA was determined in 15/18 patients with a median baseline level of 3.30 ng/mL.

**Table 1:** Clinicopathological characteristics of CRC patient cohort.

Characteristics	Patients, n (%)
**Median age (years)**	
60 (range: 28–87)	18 (100%)
**Gender**	
Male	12 (66.7%)
Female	6 (33.3%)
**ECOG**	
0	14 (77.7%)
1	3 (16.7%)
2	1 (5.6%)
**Primary tumor site**^**‡**^	
Right-sided	2 (11.1%)
Left-sided	4 (22.2%)
Rectum	12 (66.7%)
**Status of resection of primary tumor site**	
R0	17 (94.4%)
R1	1 (5.6%)
**Tumor size**	
T1	1 (5.6%)
T2	3 (16.7%)
T3	14 (77.7%)
**Nodal stage**	
N0	9 (50.0%)
N1	2 (11.1%)
N2	6 (33.3%)
Nx	1 (5.6%)
**Distant metastases**	
M0	12 (66.7%)
M1	6 (33.3%)
**Stage**^**§**^	
I	2 (11.1%)
IIA	8 (44.4%)
IIIA	1 (5.6%)
IIIC	1 (5.6%)
IVA	4 (22.2%)
IVB	2 (11.1%)
**Histological differentiation**	
G2	13 (72.2%)
G3	1 (5.6%)
Unknown	4 (22.2%)
**Lymphovascular invasion**	
L0	11 (61.1%)
L1	7 (38.9%)
**Vein invasion**	
V0	14 (77.7%)
V1	4 (22.2%)
**Perineural invasion**	
Pn0	14 (77.7%)
Pn1	3 (16.7%)
Unknown	1 (5.6%)
**Metastatic sites**^**†**^	
Lymph nodes	8 (50%)
Liver	6 (37.5%)
Lung	2 (12.5%)
**Microsatellite instability**	
MS-stable	17 (94.4%)
MSI-high	1 (5.6%)
**Therapy setting**	
Radiochemotherapy	3 (16.7%)
Chemotherapy	8 (44.4%)
Tumor after-care	7 (38.9%)
**Baseline CEA**	
Negative (< 5 ng/mL)	10 (55.5%)
Positive (≥ 5 ng/mL)	5 (27.8%)
Unknown	3 (16.7%)
Baseline CEA, ng/mL*	
3.30 (IQR: 2.00–9.50)	15 (83.3%)

*UICC* Union for International Cancer Control, *ECOG* Eastern Cooperative Oncology Group performance status, *CEA* carcinoembryonic antigen, *IQR* interquartile range.

^‡^Colon and rectum carcinomas were defined according to European standards of UICC. Right-sided colon is defined as a proximal colon region from the cecum and ascending colon to the right transverse colon. Left-sided colon is defined as a distal colon region from the left transverse colon to the descending and sigmoid colon, not including the rectum.

^§^Patients were categorized according to UICC classification (based on TNM: stage of tumor, metastases and lymph nodes) into CRC patient groups as follows: non-metastatic implies Tumor size = T1 or T2, Nodal stage = N0, Metastases stage = M0, UICC stage I or II; locally advanced implies Tumor size = T2 or T3, Nodal stage ≥ N1, Metastases stage = M0, UICC stage III; and metastatic implies Tumor size = T3, Nodal stage ≥ N1, Metastases stage = M1, UICC stage IV. Patient 19 was a special case due to the occurrence of simultaneous carcinoma of rectum (T1) and descending colon (T3), and therefore was assigned to patient group of locally advanced cancer.

^†^Metastatic sites involve repeated counting of patients because of multiple metastases.

*Data are presented as the median (interquartile range).

### cfDNA level and fragment size depend on the presence of tumor and treatment

**Figure 1:**
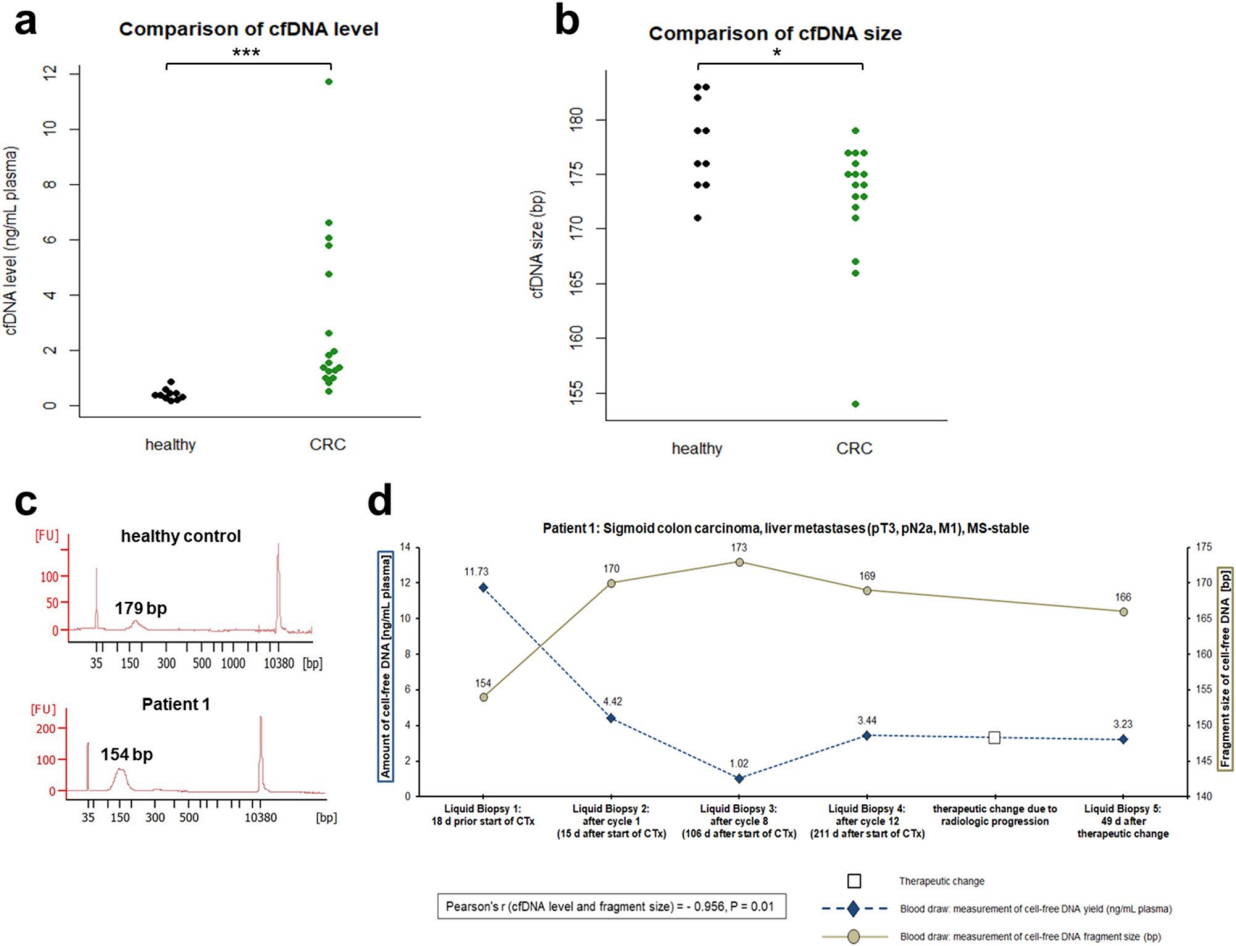
cfDNA levels and fragment sizes in CRC patients and healthy individuals. Amount in ng/mL plasma (**a**) and fragment size in bp (**b**) of cfDNA extracted from healthy individuals (n = 10) and CRC patients (n = 17). Data are presented as swarmplots and median values and significance was therefore tested using Mann–Whitney U test. cfDNA level and size were measured by using Agilent High Sensitivity DNA Kit for Bioanalyzer instrument. Representative examples of an electropherogram (fluorescence units FU plotted against fragment size in bp) from a healthy donor and metastatic patient 1 are provided (**c**). Median cfDNA levels were significantly higher and median cfDNA fragment sizes were shorter in CRC patients than in healthy individuals. Mann–Whitney U test: *P < 0.05, ***P < 0.001. (**d**) Monitoring cfDNA yield (ng/mL plasma) and cfDNA fragment size (bp) of patient 1 prior and during chemotherapy (liquid biopsies 1–5) revealed a significantly strong negative correlation between cfDNA levels and fragment sizes (Pearson’s correlation coefficient r: − 0.956, *P* = 0.01). When patient 1 showed good response to first-line chemotherapeutic treatment (FOLFIRI + cetuximab), liquid biopsies 1–3 showed a decrease of cfDNA levels and an increase of cfDNA fragment sizes. Due to radiologic disease progression in the course of further treatment, therapy regimen changed to FOLFOX + bevacizumab, while liquid biopsy 4 simultaneously revealed an increase of cfDNA yield and a decrease of cfDNA fragment size.

To study the value of quantitative and qualitative analysis of cfDNA as a biomarker in CRC, we measured levels and fragment sizes in plasma samples from 17 previously untreated CRC patients (one metastatic patient of the cohort was previously treated and thus dropped out) and 10 healthy controls by using Agilent Bioanalyzer instrument (Fig. 1a–c). Thereby, CRC patients were considered as entirety (n = 17) as well as divided into different patient groups: non-metastatic (n = 9), locally advanced (n = 3) and metastatic (n = 5). Median pretherapeutical cfDNA level of all CRC patients at baseline blood draw was 1.58 ng/mL plasma and median pretherapeutical fragment size was 174 bp. Thus, cfDNA level of CRC patients (1.58 ng/mL plasma) was as expected about fourfold higher compared to the median cfDNA level of healthy subjects (0.40 ng/mL plasma, *P* < 0.001). Besides, median cfDNA levels of the respective CRC patient groups (non-metastatic: 1.84 ng/mL plasma, *P* < 0.001; locally advanced: 1.29 ng/mL plasma, *P* < 0.05; metastatic: 1.41 ng/mL plasma, *P* < 0.001) were also significantly higher than in the healthy control group. Furthermore, the median cfDNA fragment size of all CRC patients (174 bp, *P* < 0.05) as well as those of each CRC patient group (non-metastatic: 175 bp, *P* = 0.187; locally advanced: 173 bp, *P* = 0.106; metastatic: 167 bp, *P* < 0.05) was shorter than the median cfDNA fragment size of healthy individuals (178 bp). Therefore, consideration of cfDNA level and fragment size could give an indication of the presence of a disease, and thus might be clinically relevant for diagnosis of CRC.

Next, we examined whether there is a correlation between level and fragment size of cfDNA and the course of disease as well as response to therapy. To track changes of these parameters during therapy, we collected serial blood samples from a metastatic CRC patient undergoing palliative chemotherapy (Fig. 1d). We obtained liquid biopsies prior to start of treatment and at another four time points during therapy (liquid biopsies 1–5: prior chemotherapy, after cycle 1, after cycle 8, after cycle 12, after therapeutic switch). Patient 1 was initially treated with 5-fluorouracil (5-FU), folinic acid, irinotecan (FOLFIRI), and the anti-*EGFR*-antibody cetuximab for metastasized CRC^28^ which led to a good therapy response in the next restaging after 3 months. At the same time, longitudinal cfDNA profiling from prior start of therapy until 3-month follow-up after cycle 8 (liquid biopsies 1–3) showed a decreasing cfDNA yield by approximately the factor 11 (range 1.02–11.73 ng/mL plasma), and in contrast the fragment size of cfDNA increased by 19 bp (range 154–173 bp). These observations together suggest that tumor load was shrinking. During further first-line treatment, the patient showed radiologic disease progression. Simultaneously, we detected a threefold increase of cfDNA yield (range 1.02–3.44 ng/mL plasma) and a decrease of cfDNA fragment size by 4 bp (range 169–173 bp) in liquid biopsy 4, indicating a progression of disease as well. The disease progression of patient 1 led to a therapeutic switch to 5-fluorouracil, folinic acid, oxaliplatin (FOLFOX), and the anti-*VEGF*-antibody bevacizumab. After changing therapy regimen, liquid biopsy 5 showed very similar cfDNA level and size as it was before the switch. Overall, we found a significant negative correlation between cfDNA levels and fragment sizes (Pearson’s correlation coefficient r: − 0.956, *P* = 0.01), thus a high cfDNA level is correlated to a short fragment size. Reported cfDNA levels and sizes as well as their changes during therapy suggest that quantitative and qualitative analyses of cfDNA give insights into the course of disease and therefore could be used for monitoring therapy response of CRC patients in clinical setting.

### dPCR enables up to ten times more sensitive variant detection than NGS

**Figure 2:**
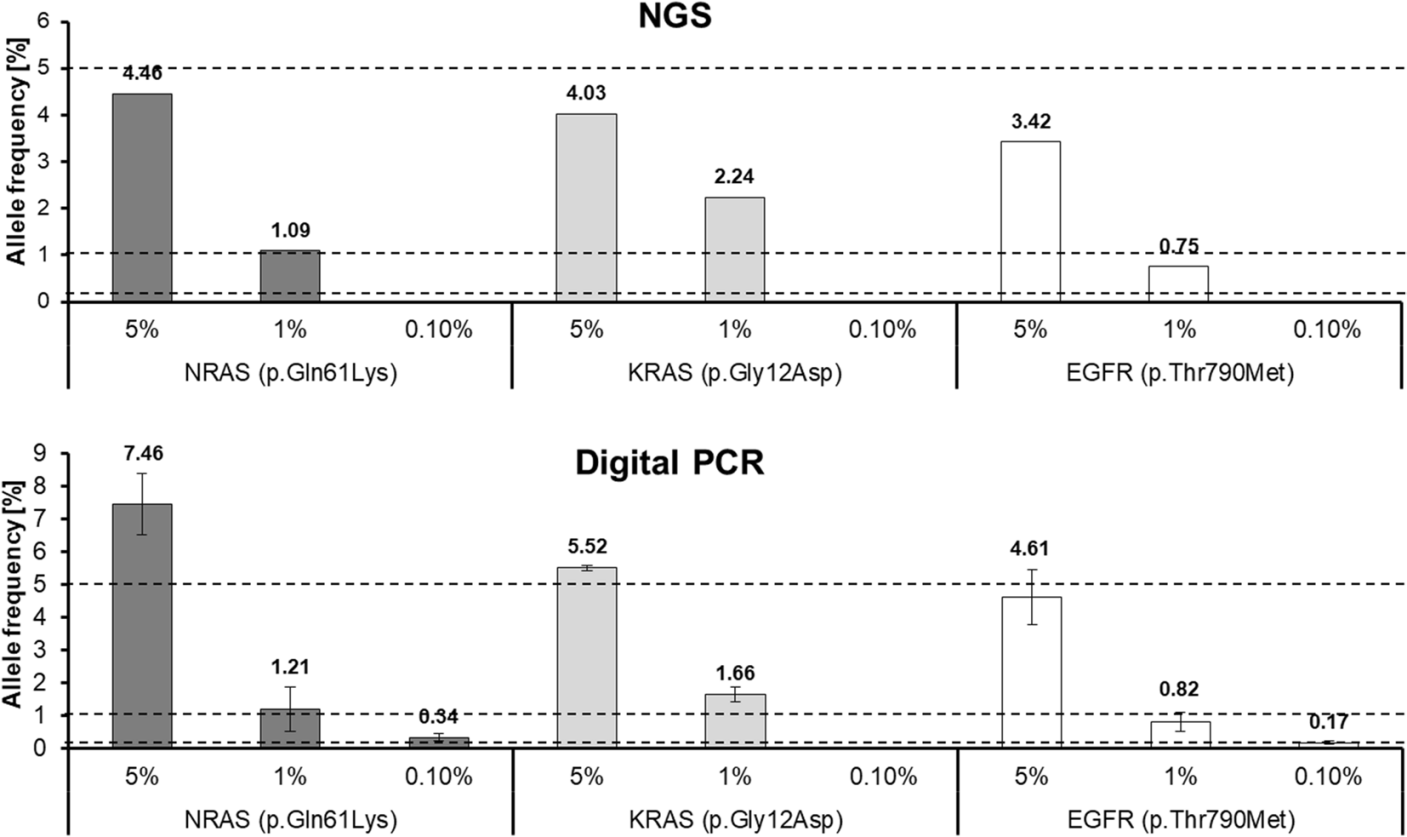
Detection limits of NGS and dPCR. Validation of applied molecular approaches was performed by using 5 ng of the Multiplex cfDNA Reference Standard Set (HD780) for analyzing with NGS (Archer Reveal ctDNA 28 Concordance Kit, n = 1) and dPCR TaqMan assays (n = 3, mean ± SD). The detection of three variants (NRAS p.Gln61Lys, KRAS p.Gly12Asp, EGFR p.Thr790Met) at given allele frequencies (5%, 1%, 0.1%) was evaluated. Dashed lines indicate expected allele frequencies provided by the manufacturer of the reference standard and columns represent frequencies measured with NGS and dPCR. NGS and dPCR enabled variant detection at 5% and 1% allele frequency, and dPCR assays additionally detected variants at allele frequencies of 0.1%.

Considering the implementation of plasma variant detection into clinical practice, it is of high importance to define standards for the molecular approaches and to validate the assays. In particular, setting detection limits is required due to the occurrence of variants predominantly at low allele frequencies to ensure the report of only true-positive variants. Evaluation of the detection limits of the applied NGS workflow (Archer Reveal ctDNA 28 Concordance Kit) and dPCR analyses (TaqMan assays) was performed by using a reference standard consisting of cell line DNA fragmented to an average size of 160 bp resembling cfDNA, with mutations (*NRAS* p.Gln61Lys, *KRAS* p.Gly12Asp, *EGFR* p.Thr790Met) at predefined allele frequencies (5%, 1%, 0.1%) (Fig. 2). NGS analysis of the reference material enabled detection of all three mutations at the expected 5% and 1% allele frequency, but none of these variants were detectable at 0.1% allele frequency. In dPCR analyses, all three variants were also detectable at the given allele frequency of 5% and 1%, and even two of these variants at 0.1% allele frequency were reproducibly measured. Altogether, NGS approach and dPCR enable variant detection up to 1% allele frequency (3/3 mutations), and dPCR additionally up to 0.1% allele frequency (2/3 mutations).

### Detection of sequence and structural variants in nuclear cfDNA is possible but input amount and read depth are limiting factors

**Figure 3:**
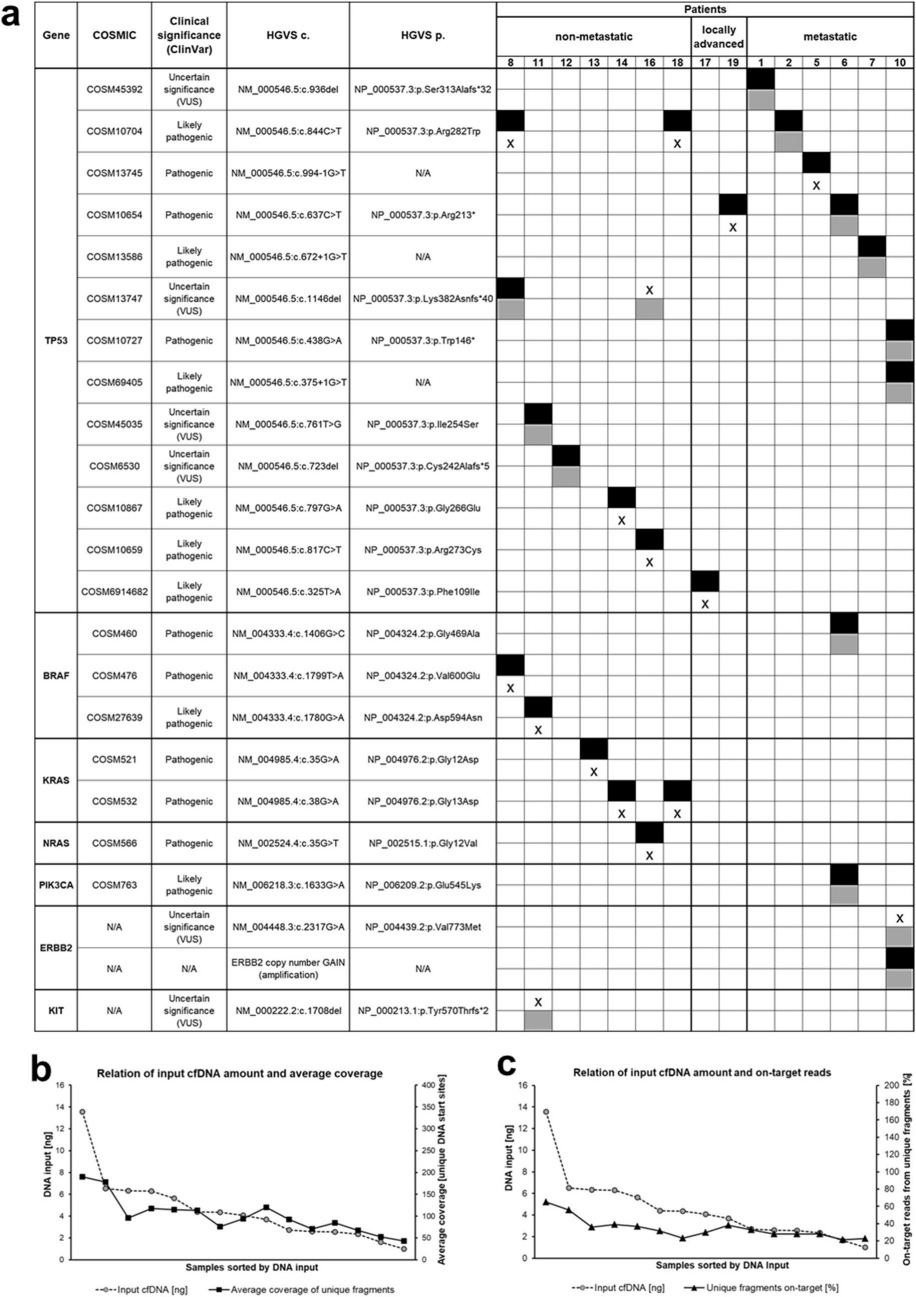
Comparative NGS analysis of nuclear DNA from tissue and plasma. (**a**) Results of NGS analysis of 15 CRC patients using Archer Reveal ctDNA 28 Concordance Kit. We subsequently excluded some patients (patient 4, 9, 15) of the CRC cohort (see “Materials and methods” section) and therefore these sample IDs are missing in the depiction. Patients were categorized as non-metastatic (n = 7), locally advanced (n = 2), and metastatic (n = 6). Black boxes indicate variants identified in FFPE tumor samples, gray boxes indicate variants identified in cfDNA from plasma and crosses indicate lacking variants. Variant allele frequencies (VAFs) are listed in Table 2. Analyzed tissue and plasma samples were except from patient 2 taken before treatment while both samples from patient 2 were taken after radiochemotherapy. Sequence variants were classified according to the recommendations of the American College of Medical Genetics and Genomics (ACMG). Overall, 25 molecular alterations were detected in FFPE DNA of which 12 variants (variant-level concordance: 48.0%) were also detectable in cfDNA of eight patients, resulting in a patient-level concordance of 53.3% (8/15). Moreover, cfDNA analysis showed additionally variants in three patients (20%; patient 10, 11 and 16). (**b**) Average coverage from unique start sites was much higher in tissue samples than in corresponding plasma samples. Furthermore, average coverage and cfDNA input amount showed a significantly strong positive correlation (Spearman’s correlation coefficient r = 0.868, *P* < 0.001). (**c**) FFPE DNA showed a much higher percentage of on-target reads from unique fragments than cfDNA. Besides, percentage of on-target reads and cfDNA input amount showed a significantly strong positive correlation (Spearman’s correlation coefficient r = 0.836, *P* < 0.001). Databases for evaluation of variants: COSMIC^67^: https://cancer.sanger.ac.uk/cosmic, ClinVar^69^: http://www.ncbi.nlm.nih.gov/clinvar, CIViC^68^: https://civicdb.org/home; c.: coding DNA reference sequence, p.: protein reference sequence, VUS**:** variant of uncertain significance, N/A: not available.

Next, we have compared NGS detection of sequence variants and CNVs from plasma to the current standard of analyzing tissue biopsies to examine the reliability of the used cfDNA workflow and to define quality thresholds for the applied molecular approaches. NGS screening of the tissue samples from 15 CRC patients of the cohort (non-metastatic: n = 7, locally advanced: n = 2, metastatic: n = 6) revealed at least one molecular alteration in eight (53.3%), and two or more in seven (46.7%) of the patients, including in total 25 molecular alterations in the FFPE DNA (Fig. 3a). Of these, 15 were missense SNVs (60.0%), 3 were deletions (12.0%), 3 were nonsense SNVs (12.0%), 3 were splice site alterations (12.0%), and 1 was a structural variant, more precisely a CNV (4.0%). Altogether, the most frequently altered gene was *TP53* (64.0%) and the mutation patterns were consistent with typically mutated genes observed in CRC, previously reported by others^29–33^. However, comparative NGS analysis of plasma DNA exhibited 12 of the 25 molecular alterations from tissue DNA, which resulted in a variant-level concordance of 48.0% (Fig. 3a). Although NGS analysis of the cfDNA reference standard has previously determined a detection limit of 1%, known variants from tissue DNA which occur below 1% allele frequency and in addition show appropriate quality metrics in cfDNA NGS analysis were also considered genuine. Average variant allele frequency (VAF) of the tissue-plasma concordant variants from FFPE DNA samples was 37.8% (range 2.0–64.2%), and 14.5% (range 0.6–59.9%) for cfDNA samples (Table 2). Moreover, in three patients (20%) cfDNA analysis revealed additionally and therefore heterogenous variants in *ERBB2*, *KIT* and *TP53* that were not detected in FFPE DNA, but all of them have uncertain significance (Fig. 3a). Furthermore, tissue-plasma concordant patients were defined as having at least one concordant variant in plasma that matched the tumor tissue results, and thus 8 of the 15 CRC patients were tissue-plasma concordant, resulting in a patient-level concordance of 53.3%. Considering the patient-level concordance with regard to the respective CRC patient groups (non-metastatic, locally advanced, metastatic), patient-level concordance reached 83.3% (5/6) in the metastatic patient group, while among the two patients with locally advanced disease none of the tissue variants were detectable in matched plasma samples and in the non-metastatic patient group concordance was solely 42.9% (3/7). This indicates an impeded variant detection in patients with lower tumor burden. Besides, patients lacking detection of tissue variants in matched cfDNA (n = 7, 46.7%) were primarily non-metastatic patients (4/7) or had locally advanced diseases (2/7). Overall, there was only one case of a metastatic patient (1/7) lacking a concordant variant from tissue in the respective plasma sample, arising presumably due to the technical fact that this patient had nearly the lowest cfDNA input amount (1.6 ng) of whole NGS analysis. Therefore, the sample consequently did not fulfill the quality metrics (coverage, on-target reads) for a reliable evaluation of variants. Thus, variant detection is critical below certain quality thresholds and leads to tissue-plasma discordance. In addition, cfDNA input amount showed overall a significantly strong positive correlation with coverage (Spearman’s correlation coefficient r = 0.868, *P* < 0.001; Fig. 3b) and percentage of on-target reads (Spearman’s correlation coefficient r = 0.836, *P* < 0.001; Fig. 3c). Furthermore, average target coverage was higher in tissue samples (average: 274) than in corresponding plasma samples (average: 101), as well as the average percentage of on-target reads (FFPE DNA: 77.8%, cfDNA: 34.4%). Accordingly, NGS analysis of cfDNA provided best results in metastatic patients and revealed that a low cfDNA input amount limits detection of molecular alterations due to lower values of coverage and on-target reads.

**Table 2:** Variant allele frequencies (VAF) of the comparative NGS analysis from nuclear DNA.

Patient	Gene	HGVS c.	HGVS p.	VAF FFPE DNA (%)	VAF cfDNA (%)
1	TP53	NM_000546.5:c.936del	NP_000537.3: p.Ser313Alafs*32	16.7	59.9
2	TP53	NM_000546.5:c.844C > T	NP_000537.3: p.Arg282Trp	59.2	5.7
5	TP53	NM_000546.5:c.994-1G > T	\	29.4	X
6	TP53	NM_000546.5:c.637C > T	NP_000537.3: p.Arg213*	64.2	3.2
	BRAF	NM_004333.4:c.1406G > C	NP_004324.2: p.Gly469Ala	58.0	3.1
	PIK3CA	NM_006218.3:c.1633G > A	NP_006209.2: p.Glu545Lys	56.5	3.4
7	TP53	NM_000546.5:c.672 + 1G > T	–	20.6	0.7
8	TP53	NM_000546.5:c.844C > T	NP_000537.3: p.Arg282Trp	27.1	X
		NM_000546.5:c.1146del	NP_000537.3: p.Lys382Asnfs*40	6.8	0.9
	BRAF	NM_004333.4:c.1799 T > A	NP_004324.2: p.Val600Glu	26.9	X
10	TP53	NM_000546.5:c.438G > A	NP_000537.3: p.Trp146*	32.8	35.4
		NM_000546.5:c.375 + 1G > T	–	28.9	31.8
	ERBB2	NM_004448.3:c.2317G > A	NP_004439.2: p.Val773Met	X	0.2
11	TP53	NM_000546.5:c.761 T > G	NP_000537.3: p.Ile254Ser	57.0	0.6
	BRAF	NM_004333.4:c.1780G > A	NP_004324.2: p.Asp594Asn	18.2	X
	KIT	NM_000222.2:c.1708del	NP_000213.1: p.Tyr570Thrfs*2	X	1.4
12	TP53	NM_000546.5:c.723del	NP_000537.3: p.Cys242Alafs*5	46.4	1.1
13	KRAS	NM_004985.4:c.35G > A	NP_004976.2: p.Gly12Asp	49.1	X
14	TP53	NM_000546.5:c.797G > A	NP_000537.3: p.Gly266Glu	63.6	X
	KRAS	NM_004985.4:c.38G > A	NP_004976.2: p.Gly13Asp	42.8	X
16	TP53	NM_000546.5:c.817C > T	NP_000537.3: p.Arg273Cys	49.8	X
		NM_000546.5:c.1146del	NP_000537.3: p.Lys382Asnfs*40	X	3.3
	NRAS	NM_002524.4:c.35G > T	NP_004976.2: p.Gly12Asp	47.4	X
17	TP53	NM_000546.5:c.325 T > A	NP_000537.3: p.Phe109Ile	39.2	X
18	TP53	NM_000546.5:c.844C > T	NP_000537.3: p.Arg282Trp	4.4	X
	KRAS	NM_004985.4:c.38G > A	NP_004976.2: p.Gly13Asp	2.0	X
19	TP53	NM_000546.5:c.637C > T	NP_000537.3: p.Arg213*	60.1	X

X indicate variants which were not detected in the respective DNA from tissue or plasma. c.: coding DNA reference sequence, p.: protein reference sequence.

**Figure 4:**
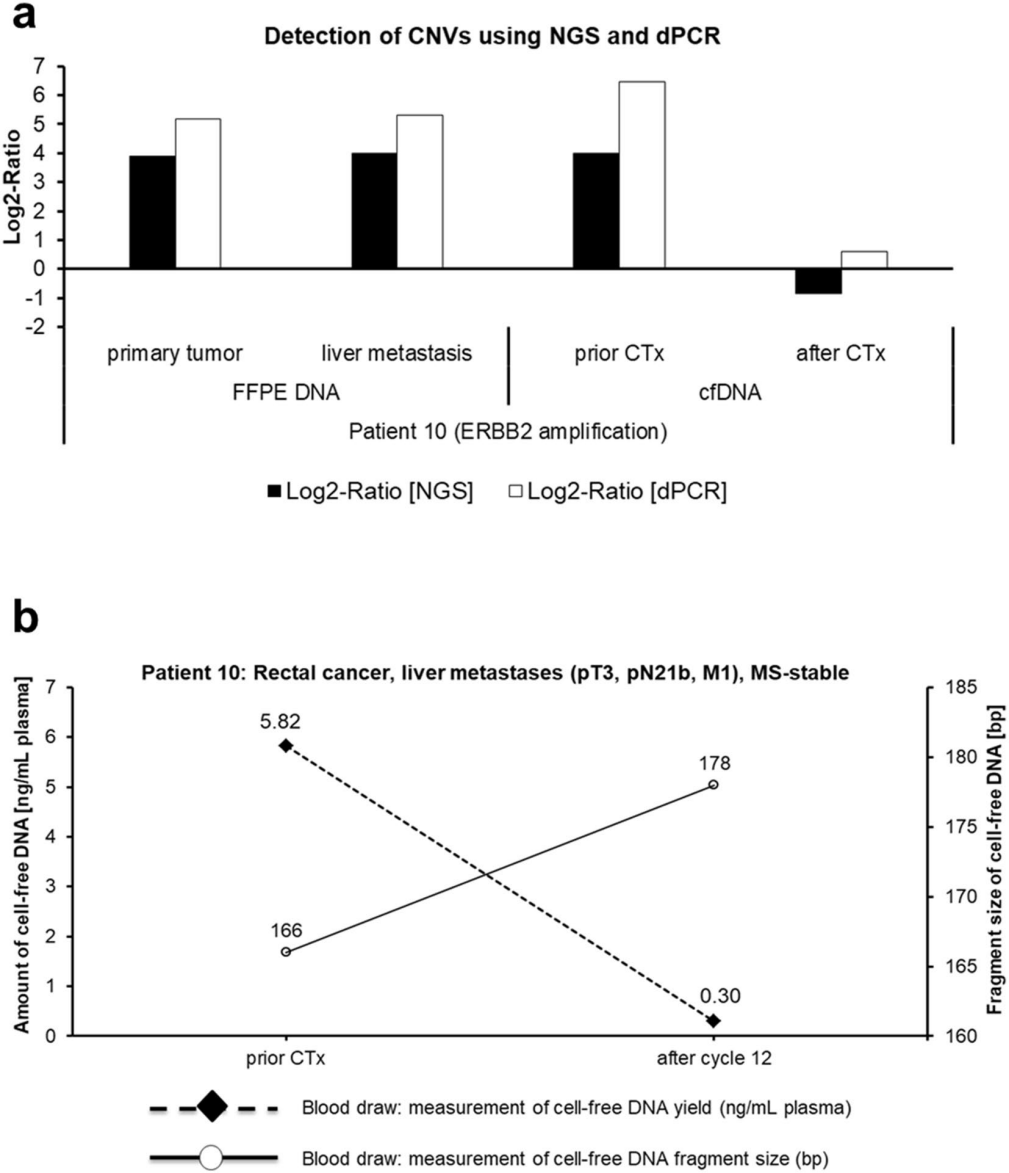
Validation of copy number variation analysis using NGS and dPCR. (**a**) Comparison of Log2-Ratios from NGS using Archer Reveal ctDNA 28 Concordance Kit and dPCR TaqMan analysis showed good concordance. Gain of ERRB2 was detectable in tissue sample of primary tumor and liver metastasis, and also in cfDNA before chemotherapy (FOLFIRI + cetuximab), but not in cfDNA after it. (**b**) Course of amount and fragment size of cfDNA prior and after chemotherapeutic treatment of patient 10 was in agreement with variant detection. CTx: chemotherapy.

Apart from sequence variants, NGS analysis revealed a structural variant of the oncogene *ERBB2* (also called *HER2*) in both FFPE DNA and cfDNA of metastatic patient 10 (Fig. 3a). To further validate the NGS results of CNV status of patient 10, we performed dPCR analysis with a copy number TaqMan assay detecting this structural variant. Herein, Log2-Ratios from NGS approach and dPCR analyses showed good concordance (Fig. 4a). Consistently with both approaches, *ERBB2* amplification could be detected in tissue samples of primary tumor and liver metastasis, and in cfDNA before chemotherapeutic treatment, but not after chemotherapy. This is in accordance with the detected amount and fragment size of cfDNA from patient 10, as cfDNA level decreased by almost 20 times and fragment size increased by 10 bp from the time before chemotherapy towards the end of it (Fig. 4b). In relation to this, detected SNVs in *TP53* gene of patient 10 (Fig. 3a) were also solely detectable in cfDNA before chemotherapy, altogether indicating an appropriate therapy response. Overall, the results of orthogonal validation of NGS approach by dPCR showed a strong positive correlation (Spearman’s correlation coefficient: r = 0.949, *P* = 0.05; Supplementary Fig. S1), demonstrating the robust ability to detect CNVs in cfDNA by using both NGS and dPCR.

### Variant detection in mitochondrial cfDNA is difficult because of lower plasma copy number in CRC patients than in healthy controls

**Figure 5:**
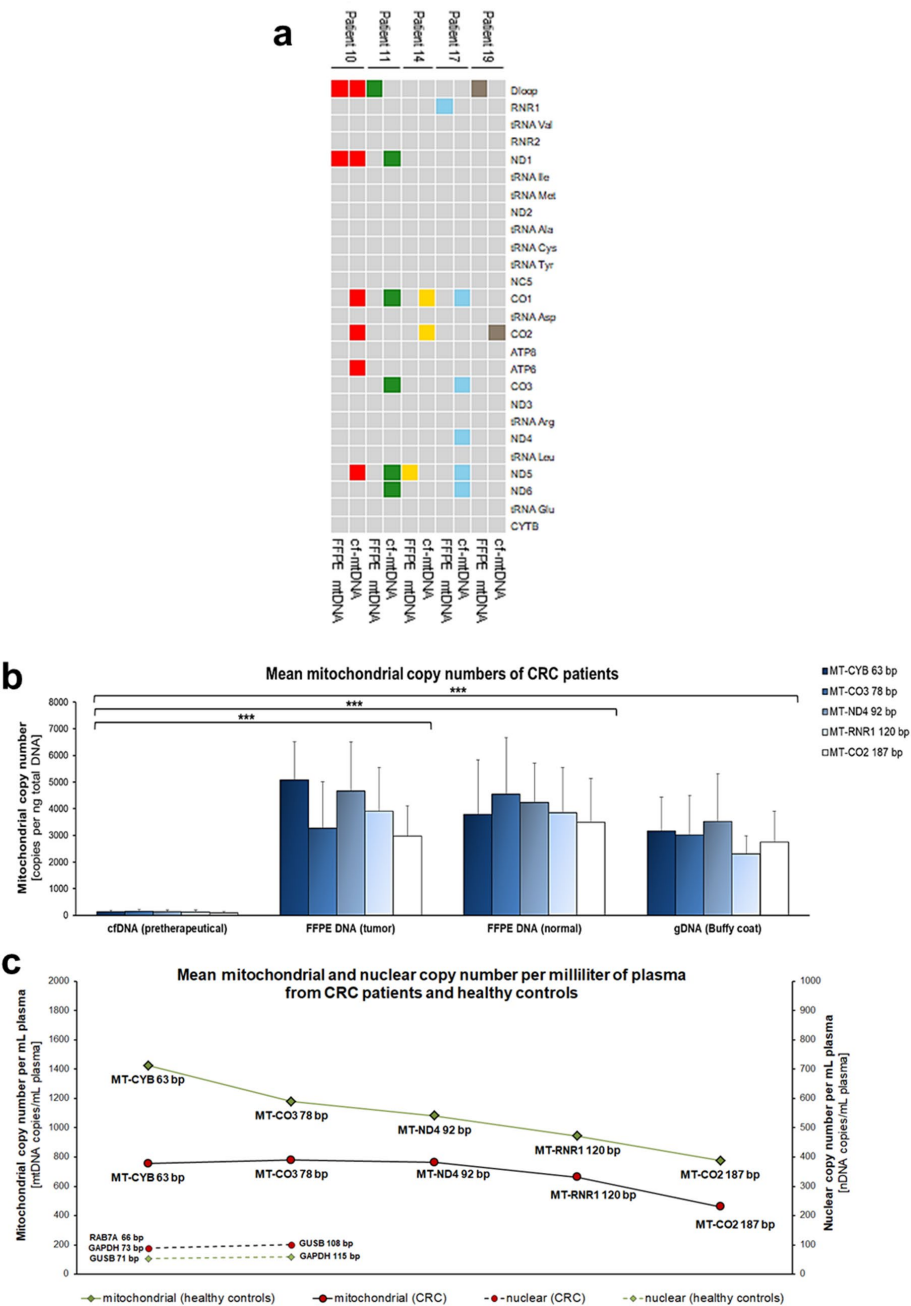
Mitochondrial variants and copy numbers in tissue and plasma. (**a**) Heatmap showing results of variant detection in five CRC patients using QIAseq Targeted Panel DHS-105Z covering whole mitochondrial genome. Depicted variants are non-coding or non-synonymous and were categorized according to their occurrence in the different loci of mitochondrial genome. Colors represent variants from matched tissue and plasma samples originating from one patient. Overall, we found eight tumor-specific variants in tissue FFPE DNA of which two (25%) were also detectable in cf-mtDNA of patient 10 (patient-level concordance: 20%). (**b**) Column graph showing average mitochondrial copy numbers (mtDNA copies per 1 ng of applied DNA) of prethereapeutical cf-mtDNA, mtDNA from tumor and adjacent normal tissue, and from buffy coat of in total six CRC patients that had no distant metastases. Copy numbers were determined with dPCR TaqMan assays of varying amplicon length for mitochondrial DNA (*MT-CYB* 63 bp, *MT-CO3* 78 bp, *MT-ND4* 92 bp, *MT-RNR1* 120 bp, *MT-CO2* 187 bp) expressed as the mean ± SD from all patients and the respective assay. Comparison revealed that cf-mtDNA (125 ± 66) had a significantly lower copy number than mtDNA from tumor (3979 ± 1675) and normal (3982 ± 1723) tissue and from buffy coat (2949 ± 1301). Welch’s t-Test ***P < 0.001 (**c**) Comparison of average mitochondrial and nuclear plasma concentration (copy number per mL plasma) of CRC patients (n = 6) and healthy controls (n = 7) presented as the mean from all patients and the respective assay. Nuclear assays are combined to their fragment sizes, from short (*RAB7A* 66 bp, *GUSB* 71 bp, *GAPDH* 73 bp,) to long (*GUSB* 108 bp, *GAPDH* 115 bp). Average mitochondrial plasma concentration was significantly higher than average nuclear plasma concentration in CRC patients (cf-mtDNA: 684 ± 357; cf-nDNA: 93 ± 48; Welch’s t-test: *P* < 0.001) and healthy controls (cf-mtDNA: 1081 ± 780; cf-nDNA: 55 ± 19; Mann–Whitney U test: *P* < 0.01). Surprisingly, mitochondrial plasma concentration was higher in healthy controls than in CRC patients (healthy: 1081 ± 780; CRC: 684 ± 357; student’s t-test: *P* < 0.05). Overall, a decreasing copy number and therefore fragment size was noticeably in all individuals.

Addressing the issue of low input amount and the necessity of higher sensitivity for cfDNA analysis, we analyzed mitochondrial DNA (mtDNA). Here, we compared variant detection in tissue and plasma mtDNA and examined properties of cell-free nuclear DNA (cf-nDNA) and cell-free mitochondrial DNA (cf-mtDNA) in CRC patients and healthy controls with regard to implement cf-mtDNA as a potential biomarker for CRC. Sequencing of mtDNA from tumor tissue (n = 5) revealed eight tumor-specific molecular alterations with at least one variant in each patient and an average VAF of 26.2% (Fig. 5a). Of these eight mtDNA variants present in tumor specimens, only two (variant-level concordance: 25%) were found in matched cf-mtDNA from plasma samples, resulting in a patient-level concordance of only 20% and therefore showing even a lower concordance than NGS analysis of nuclear cfDNA. Moreover, cf-mtDNA revealed 31 additional variants (Supplementary Table S3) which were not detected in the corresponding tissues of the patients and thus are of unknown tissue origin. These results indicate that detection of tumor-specific mitochondrial variants in cfDNA is possible, but apparently only in patients with high tumor burden, such as metastatic patient 10 as the only case of detectable tissue mtDNA variants in matched plasma cf-mtDNA (Fig. 5a). Patient 10 previously showed a high cfDNA level (Fig. 4b) and a high frequency of tumor-specific sequence variants (Fig. 3a) and CNVs (Fig. 4a), indicating a high tumor load in plasma at time of blood sampling. Furthermore, patient 10 had the highest input amount of cfDNA and this resulted in the highest percentage of reads owning unique molecular identifiers, which are significantly correlated to each other (Spearman’s correlation coefficient r = 0.975, *P* < 0.01; Supplementary Fig. S2). This demonstrates, as already described for sequencing of cf-nDNA (Fig. 3b,c), the technical influence of cfDNA input amount on variant detection from plasma. Overall, variant detection in mitochondrial cfDNA revealed unexpected difficulties, and thus showed no considerable advantages over cfDNA analysis from nucleus.

The challenging detection of tumor-specific mitochondrial tissue variants in corresponding plasma samples was very surprising due to the actually higher copy number of mtDNA. To verify the reasons for these difficulties, we compared the copy numbers of mtDNA from tumor and adjacent normal tissue and from pretherapeutical plasma and buffy coat samples from in total six patients of the CRC study cohort. An overview of all individual copy numbers is provided in Supplementary Table S5. Comparison of average mtDNA copy number (mtDNA copies per 1 ng of total DNA; Fig. 5b) calculated from all mitochondrial assays of the six CRC patients revealed that mtDNA has a significantly lower copy number in plasma (mean 125 copies) than in the buffy coat (mean 2949 copies, *P* < 0.001), normal (mean 3982 copies, *P* < 0.001) and tumor tissue (mean 3979 copies, *P* < 0.001). This resulted in an average mtDNA copy number over 30 times higher in tumor tissue compared to plasma. Furthermore, we investigated mitochondrial and nuclear plasma copy numbers (copy number per milliliter of plasma) from CRC patients and healthy controls (Fig. 5c). Although average mitochondrial plasma copy number in CRC patients was significantly higher than nuclear plasma copy number (cf-mtDNA: mean 684 copies/mL plasma; cf-nDNA: mean 93 copies/mL plasma; *P* < 0.001), the mitochondrial plasma concentration was in total surprisingly lower in CRC patients than in healthy individuals (CRC: mean 684 copies/mL plasma, healthy: mean 1081 copies/mL plasma; *P* < 0.05), therefore reducing the intended effect of higher sensitivity for NGS approach. Moreover, mitochondrial plasma concentration was approximately twice as high when running the assay with the shortest amplicon size (*MT-CYB* 63 bp) compared to the assay with the longest amplicon size (*MT-CO2* 187 bp) (Fig. 5c). This resulted in an overall decrease of 45.5% in healthy individuals (decrease 648 copies/mL plasma) and 38.9% in CRC patients (decrease 293 copies/mL plasma), indicating the existence of a crucial proportion of short fragments in plasma mtDNA. Overall, the detection of mitochondrial variants in cfDNA using NGS approach might be affected for various technical and biological reasons. First, there generally is a much lower mitochondrial copy number in plasma than in tissue. Secondly, mitochondrial copy number is lower in CRC patients than in healthy individuals. Lastly, our results indicate that cf-mtDNA might be even shorter than cfDNA from nucleus.

## Discussion

In this study, we assessed the clinical value of several liquid biopsy applications for potential use in routine diagnostic and surveillance of CRC patients. An initial objective of the current study was the measurement of baseline cfDNA levels and fragment sizes. Consistent with previous results, the present study revealed higher cfDNA levels in cancer patients than in healthy individuals that additionally varied in the different clinicopathological groups of CRC patients^9,15,18,34^. These differences between healthy subjects and cancer patients as well as the variabilities among the CRC patient groups could, apart from the presence of a tumor, result from biologic and physiologic factors that affect the release mechanism and the clearance of cfDNA^35–37^. Furthermore, some studies have shown noticeable differences in tumor-derived ctDNA levels between patients even with the same cancer type and disease stage, resulting in an individually varying fraction of ctDNA from total cfDNA^6,11,38^. The mechanism of cfDNA release also has an impact on cfDNA fragment size, as presented by the “ladder” pattern of cfDNA because of its apoptotic origin^8,9^. The present results corroborate previous findings that length distribution of cfDNA in cancer patients exhibits a more fragmented pattern than in healthy subjects^13,39,40^. Interestingly, longitudinal analysis of plasma samples from metastatic patient 1 demonstrated very short fragments before initiation of chemotherapy with a shift to longer fragments during chemotherapy. This emergence of longer wildtype cfDNA fragments was accompanied by a decreasing cfDNA level during therapy, indicating a shrinking tumoral bulk and an appropriate therapy response. It has been demonstrated, that cfDNA levels in CRC patients correlate with changes in tumor burden and treatment response^5,41^. Moreover, several studies have shown that cfDNA levels often provide a more sensitive and specific indication of real-time tumor burden in response to therapy than standard serum tumor markers in current clinical use (e.g. CEA)^5,42^. However, most of the studies only considered cfDNA levels and not longitudinal analysis of cfDNA size, although it is difficult to assess therapy response solely based on one parameter. In contrast, our study demonstrates that measurements of both, cfDNA level and size, can serve as reliable markers in clinical practice of CRC patients, not only to discriminate between healthy individuals and cancer patients (alone or in combination with blood protein tumor markers), but also to assess therapy response when performed as serial cfDNA level and size measurements to complement the information available from radiologic imaging.

In the present study, we observed a good patient-level concordance (53.3%) from NGS analysis of nuclear tissue and plasma DNA. This value is consistent with previously reported NGS detection rates in CRC patients (39–85.9%)^43–46^. However, in almost half of the patients (predominantly non-metastatic patients) we could not detect any of the tissue variants in matched plasma cfDNA, indicating that tissue analysis is particularly essential in non-metastatic CRC patients compared to metastatic patients. There are several possible biological and technical explanations for failing detection of variants in cfDNA and thus generating false-negative results. Firstly, it may be assumed that the number of available mutant molecules in plasma of the patients was too low for detection. In a landmark study of 640 patients with different types and stages of cancer, including CRC, quantification of individual tumor-associated mutations in each patient revealed that stage I patients had solely less than 10 mutant copies per 5 mL plasma, while patients with advanced cancers (stage IV) had more than 100 mutant copies per 5 mL plasma^6^. Related to these insights, the non-metastatic CRC patients of our study cohort who failed the detection of variants in cfDNA probably provided only a small fraction of copies carrying the mutation because they were at lower tumor stages (stage I-II) and median volume of used plasma was even lower than 5 mL, thus impeding variant detection in plasma. Secondly, variants in cfDNA occur mainly at very low allele frequencies and often < 1%^5,11,47^. Thus, the sensitivity of NGS approach might have been too low for detecting those less frequent mutations. The possibility of detecting variants below 1% is mostly only provided if sequencing of the tissue DNA was performed first to search for known variants in cfDNA. Nevertheless, such variants must fulfill certain quality parameters (e.g. coverage) to be considered as true-positive variants. The achievable sensitivity of cfDNA assays is dependent on several factors, including sampling volume, cfDNA input amount, and the fraction of tumor-derived ctDNA. Concerning the present study, targeted input amount of 5 ng DNA corresponds to 1500 copies and therefore to 750 diploid genome equivalents (DNA amount corresponding to a diploid genome of a cell) which in theory leads to a sensitivity of ~ 0.07%^16,48^. However, evaluation of the detection limits of the applied molecular workflows demonstrated that such calculated sensitivities cannot easily be translated into practice as presented by the determined lower detection limits of NGS (1%) and dPCR (0.1%). As discussed, this is evoked by the biological and physiological variabilities and thus poses a considerable challenge for accurate and reproducible variant detection in plasma. Furthermore, it can also be assumed that observed discordance of tissue and plasma analysis is affected by technical assay variation, as we have demonstrated by the correlation of cfDNA input amount and the quality metrics coverage and on-target reads, and therefore the impact on confidence of plasma mutation detection.

Another finding of the present study was that plasma NGS analysis revealed additional variants in cfDNA of three cases (20%), indicating that liquid biopsies enable in principle insights into tumor heterogeneity. Such mutations could arise due to intratumoral heterogeneity^49^ or metastasis-private events such as chromothripsis which has been shown to be prevalent in CRC^50^. Chromothripsis is defined as a single catastrophic phenomenon in which one or a few chromosomes in a cancer cell harbor clustered rearrangements and therefore oncogene amplifications. Indeed, NGS and dPCR analysis of metastatic patient 10 revealed an amplification of the oncogene *ERBB2* (*HER2*). The examination of *ERBB2* copy numbers is of emerging diagnostic value, because amplifications of *ERBB2* are a potential therapeutic target for metastatic CRC and have shown to be involved in acquired resistance to anti-*EGFR* antibodies^51,52^. Therefore, one interesting clinically relevant findings of the present study is that we were able to reliably detect this amplification in ctDNA of patient 10. Moreover, in a recent study with known *ERBB2*-positive metastatic CRC patients the use of ctDNA also showed accurate determination of *ERBB2* copy numbers and therefore was as reliable as tissue-based genotyping^53^. These results emphasize that liquid biopsies might be performed as a minimally invasive alternative to tissue-based detection of CNVs for assessment of therapy response or application of another therapy regimen with a better response.

Next, we hypothesized that variants in cf-mtDNA would be more easily detectable as those in cf-nDNA due to higher copy number per cell and thus would require less sensitive applications to detect them. Overall, our results of comparative NGS analysis of mtDNA are consistent with a recently published study, also reporting limited ability in tracing tumor-specific cf-mtDNA variants by sequencing^54^. Most of the variants they identified from tumor tissue were undetectable in matched plasma mtDNA, resulting in a variant-level concordance (17%) even lower than ours (25%). Although in the present study NGS analysis of mitochondrial cfDNA revealed many additional variants, it can be assumed that these variants did not occur due to tumor heterogeneity and thus are not tumor-specific for CRC, because detection of the respective tumor tissue variants in matched cf-mtDNA was failing. The substantial discordance we observed between tissue and plasma mtDNA probably emerged from heteroplasmy, representing coexistence of mutated and wildtype mtDNA molecules in the same cell, tissue or organ^55^. The heteroplasmic variant pattern can differ within the tissues of an individual, often constituting heteroplasmic patient-specific variants from normal tissue but not tumor-specific heteroplasmic mtDNA variants^56^. However, cf-mtDNA, which is released from different cell types into circulation, represents the entirety of variants and therefore arising variant pattern is dependent on tissue origin of cf-mtDNA^56,57^.

Currently, it is still unclear why variant detection from plasma mtDNA is that challenging. There are several factors that should be considered: first, the variable mtDNA copy number of each cell can evoke that cells of non-tumor tissues have a higher mtDNA abundance than tumor cells. Thus, it might be that primarily non-tumor tissues are shedding mtDNA into blood circulation opposed to less frequent release of tumor-derived mtDNA. This leads to an accumulation of non-tumor variants and therefore an impeded detection of tumor-specific plasma mtDNA mutations due to a high wildtype background. Furthermore, a short fragment size of cf-mtDNA has been noticed by us and others which can be partially ascribed to the lack of nucleosome-associated histone proteins, resulting in a more fragmented pattern than its nuclear counterpart^8,58,59^. Another possible explanation is that mtDNA might not be present in a cell-free form. A recent study described the occurrence of intact cell-free mitochondria with extracellular full-length mtDNA in blood^60^. Hence, it is possible that the mitochondrial fraction we have examined with the applied molecular approaches was not as expected primarily compound of short fragments but rather of long mtDNA which was not caught with the used techniques. Therefore, optimized protocols, such as size selection of cf-mtDNA fragments or an alternative method for library preparation like single-strand DNA libraries, are possible solutions for a promising outcome concerning analysis of cf-mtDNA. So far, there have been only a few studies investigating plasma mtDNA in CRC patients. In consistency with our results, those studies reported that mtDNA copy number is lower in plasma samples from CRC patients than in healthy subjects, also hampering detection of tumor-specific variants in plasma^59,61^. In many cancers, a reduction of mtDNA copy number is a common feature^62^. In addition, it has been reported that patients with low mtDNA content have increased benefit from chemotherapeutic treatment due to higher susceptibility to mitochondrial damage^63^. Therefore, in clinical settings it is conceivable that determination of mtDNA copy numbers may be helpful for prospective assessment of therapy response. However, this study included only a small sample size. For a comprehensive use of liquid biopsies in clinical practice further studies of paired tumor and plasma samples are necessary to evaluate the diagnostic accuracy of the analytical platforms, especially for variants with allele frequencies close to the limit of detection which should be interpreted with caution. Besides, defining standards for liquid biopsy approaches is still an ongoing process. Furthermore, the discordance of mutations detected from tumor tissue and plasma cfDNA reported in many studies is still one of the major challenges for application of liquid biopsies.

## Materials and methods

### Study design and patient cohort

Between July 2017 and August 2020, we enrolled 22 colorectal cancer (CRC) patients that were treated surgically and/or with chemotherapy at the University Medical Center Mainz (Germany). All patients had a pathological diagnosis of primary colorectal adenocarcinoma. Of the enrolled 22 CRC patients, four patients (patient 4, 9, 15, 20) were subsequently excluded for one of the following reasons: lack of pretherapeutical tissue and/or plasma, inflammatory disease, or no detectable cfDNA. Thus, the final study cohort consisted of 18 patients with non-metastatic (n = 9), locally advanced (n = 3) or metastatic (n = 6) CRC. Biological material, including DNA from biopsied or resected tissue specimen and cfDNA from plasma, was predominantly (in 17/18 patients) collected before any treatment for comparison of tissue and plasma mutation status and copy numbers. Levels of the protein tumor marker carcinoembryonic antigen (CEA) were also measured before treatment by the local diagnostic laboratory with CEA concentrations of < 5 ng/mL considered normal. We also included a control group of 10 apparently healthy individuals with no current cancer diagnosis. Of these healthy controls, 80% were female and median age was 31 years (range 23–56 years).

### Sample collection and processing

Formalin-fixed paraffin-embedded (FFPE) DNA from diagnostic biopsied or resected tumor and adjacent normal tissues was extracted from ~ 5 µm thick tissue sections using QIAamp DNA FFPE Tissue Kit (Qiagen, Hilden, Germany). FFPE DNA quantification was performed using Qubit 1.0 Fluorometer (Thermo Fisher Scientific, Dreieich, Germany). Venous blood from healthy donors and CRC patients was taken with a blood collection set with preattached holder (Becton Dickinson, Heidelberg, Germany) and PAXgene Blood ccfDNA Tubes (Qiagen). From all individuals, we collected two tubes with a maximum volume of 10 mL each. After blood drawing, tubes were immediately inverted and directly processed. Whole-blood samples were centrifuged at 2.000 × g for 10 min at 4 °C and plasma was transferred to a fresh tube and was centrifuged a second time at 16.000 × g for 10 min at 4 °C. Plasma was then used for extraction of cfDNA with QIAamp MinElute ccfDNA Midi Kit (Qiagen) and buffy coat layer containing the white blood cells was used for extraction of genomic DNA (gDNA) using QIAmp DNA Blood Mini Kit (Qiagen). Concentration of gDNA was measured with Qubit 1.0 Fluorometer (Thermo Fisher Scientific). Quality and quantity of extracted cfDNA was assessed via electropherograms by using Agilent High Sensitivity DNA Kit for 2100 Bioanalyzer instrument (Agilent Technologies, Waldbronn, Germany). Data were analyzed with 2100 Expert Software version B.02.08 (Agilent Technologies). cfDNA fragment size was defined as the average size of the main peak in the electropherogram and cfDNA level was calculated as the area under the main peak.

### Molecular analysis of nuclear DNA

For comparison of tissue and plasma mutation status and copy numbers, FFPE DNA and cfDNA from CRC patients with sufficient material (n = 15) were analyzed for somatic mutations using the targeted NGS panel Reveal ctDNA 28 Concordance Kit (ArcherDx, Boulder, USA) including 28 genes (*ALK, AKT1, AR, BRAF, CTNNB1, DDR2, EGFR, ERBB2, ESR1, FGFR1, HRAS, IDH1, IDH2, KIT, KRAS, MAP2K1, MAP2K2, MET, NRAS, NTRK1, NTRK3, PIK3CA, PDGFRA, RET, ROS1, SMAD4, MTOR, TP53*). This panel is based on anchored multiplex PCR (AMP), a target enrichment method that uses unidirectional gene-specific primers and molecular barcoded (MBC) adapters for amplification^20^. Molecular barcoding identifies reads originating from the same molecule, correcting for PCR or sequencing errors^48,64–66^. The panel enables detection of sequence variants and calling of copy number variations (CNV) by counting the uniquely tagged molecules to deduce the copy number from each target region in comparison to the baseline of normal samples. For workflow validation and evaluation of detection limit, we used 5 ng of the commercially available Multiplex I cfDNA Reference Standard Set HD780 (Horizon Discovery, Cambridge, UK). For generating NGS libraries, average input amount was 4.5 ng (range 1.0–13.6 ng) for cfDNA and 184.5 ng (range 12.9–250 ng) for FFPE DNA. Libraries were quantified using Qubit 1.0 Fluorometer (Thermo Fisher Scientific) and diluted to a concentration of 4 nM. Quality control of generated libraries was performed by using 2100 Bioanalyzer Instrument (Agilent Technologies). All libraries were denatured by adding 0.2 nM NaOH and diluted to 13 pM for FFPE DNA and 10 pM for cfDNA with hybridization buffer from Illumina (San Diego, USA). The number of pooled samples was adapted to type of library to ensure FFPE DNA libraries obtain 1 million reads and cfDNA libraries 5 million reads per sample. Sequencing was performed on MiSeq instrument using MiSeq Reagent Kit v3 from Illumina and paired-end sequencing with 2 × 151 bp reads. FASTQ files were processed using Archer Analysis Software Version 6.2.3 (ArcherDx) with the appropriate SNV and CNV analysis workflow. For analysis of cfDNA, advanced settings of a read depth normalization of 10,000,000 reads, specifying the number of reads for randomly subsampling, and a variant downstream region of interest size of 150 bases, which defines the number of bases downstream of the gene-specific primers that are considered when searching for variants, were used. The software provides all secondary analysis (read trimming, de-duplication, error correction, alignment, mutation calling). Results are presented relative to hg19 (GRCh37). Molecular alterations were evaluated on entries in the cancer-specific variant databases COSMIC^67^ and CIViC^68^ as well as the variant interpretation database ClinVar^69^. Sequence variants were classified according to the recommendations of the American College of Medical Genetics and Genomics (ACMG). Coverage was defined as the reads from unique start sites in each target region with a quality control threshold of 50. All sequencing data of nuclear DNA are listed in Supplementary Table S1.

Evaluation of detection limit of dPCR (QuantStudio 3D Digital PCR System, Thermo Fisher Scientific) was performed by using 5 ng of the Multiplex I cfDNA Reference Standard Set HD780 (Horizon Discovery) and TaqMan dPCR assays from Thermo Fisher Scientific (*NRAS* p.Gln61Lys, assay ID: Hs000000079_rm; *KRAS* p.Gly12Asp, assay ID: Hs000000051_rm; *EGFR* p.Thr790Met, assay ID: Hs000000029_rm). For validation of copy number status detected with NGS workflow, we used a TaqMan copy number assay for *ERBB2* (Hs00450668_cn, Thermo Fisher Scientific). dPCR analysis enables an absolute copy number quantitation of gene of interest which is then normalized to a reference gene known to be present in two copies in a diploid genome. For each dPCR reaction, copy number assay of the target gene and the reference gene *RNase P* (assay ID: 4,403,326, Thermo Fisher Scientific) were run simultaneously with 1 ng FFPE DNA from tumor and matched normal tissue and with cfDNA from liquid biopsies of *ERBB2*-positive patient 10. dPCR was performed using QuantStudio 3D Digital PCR Master Mix and 3D PCR 20 K Chip Kit version 2 on a flat block thermocycler (ProFlex) from Thermo Fisher Scientific. Setup of dPCR reactions were as follows: hot start at 96 °C for 10 min, denaturation at 98 °C for 30 s, annealing/extension at 60 °C for 2 min for a total of 39 cycles, followed by a final extension step at 60 °C for 2 min. Data were analyzed with QuantStudio 3D AnalysisSuite Cloud Software version 3.2 (Thermo Fisher Scientific).

### Molecular analysis of mitochondrial DNA

Mitochondrial DNA (mtDNA) of pretherapeutical tissue and plasma was analyzed by NGS for variant detection and by dPCR for determination of copy numbers. For excluding variants arising from clonal hematopoiesis as well as constitutional polymorphisms and as a control for copy numbers, we analyzed FFPE DNA from tumor and tumor-adjacent normal tissue (intestinal mucosa) as well as cfDNA from plasma and the corresponding leukocyte DNA from buffy coat. NGS libraries for variant detection were constructed with the QIAseq Targeted DNA Panel-Human Mitochondria Panel (DHS-105Z; Qiagen) covering the whole mitochondrial genome. This NGS panel is based on AMP as well and uses unique molecular identifiers (UMIs) to better differentiate NGS artifacts from real mutations at very low allele fractions. For generating cfDNA libraries, the enzymatic fragmentation was inhibited. DNA input amount of tissue samples (n = 10) and buffy coat samples (n = 4) was 100 ng, and average input amount of cfDNA samples (n = 5) was 4.7 ng (range 0.5–10.0 ng). Quantification, quality control, dilution, and sequencing of the libraries were performed as described above for NGS analysis of nuclear DNA, but final concentration of all mtDNA li-braries was 10 pM. Sequencing was carried out to ensure that buffy coat DNA libraries had 500,000 reads, FFPE DNA libraries 1 million reads, and cfDNA libraries 5 million reads. FASTQ files were processed using CLC Genomics Workbench version 12.0 (Qiagen) with a customized data analysis workflow (Supplementary Table S2). Reads were aligned to Revised Cambridge Reference Sequence (rCRS) of the Human Mitochondrial DNA (NC_012920). Mitochondrial variants were evaluated on entries of the database MITOMAP^70^. Quality threshold of coverage was 100. Supplementary Table S3 gives an overview of all mitochondrial sequencing data.

Determination of copy numbers was performed using dPCR (QuantStudio 3D Digital PCR System, Thermo Fisher Scientific) with customized TaqMan assays of varying amplicon length for mitochondrial DNA (*MT-CYB* 63 bp, *MT-CO3* 78 bp, *MT-ND4* 92 bp, *MT-RNR1* 120 bp, *MT-CO2* 187 bp) and nuclear DNA (*GAPDH* 73 bp, *GAPDH* 115 bp, *GUSB* 71 bp, *GUSB* 108 bp, *RAB7A* 66 bp). Panel of amplicons with various sizes was designed using Primer 3 (http://primer3.ut.ee) and assays were manufactured by Integrated DNA Technologies (Interleuvenlaan, Belgium). mtDNA assays contained a FAM dye labeled minor groove binding probe and nuclear DNA assays contained a HEX dye-labeled TAMRA probe. TaqMan assays were run simultaneously in a duplex dPCR reaction. Sequences of primers and probes and appropriate ratios are listed in Supplementary Table S4. TaqMan assays (0.75 µl each) were mixed with 7.5 µl QuantStudio 3D Digital PCR Master Mix and variable volumes of DNA and H_2_O to a final volume of 15 µl, of which 14.5 µl were put on a 3D PCR 20 K Chip version 2 and run on a flat block thermocycler (ProFlex) from Thermo Fisher Scientific with following PCR conditions: hot start at 96 °C for 10 min, denaturation at 98 °C for 30 s, annealing/extension at 58 °C for 2 min for a total of 39 cycles, followed by a final extension step at 58 °C for 2 min. Data were analyzed with QuantStudio 3D AnalysisSuite Cloud Software version 3.2 (Thermo Fisher Scientific). Copy number analysis was performed with in total 12 tissue (tumor and normal), and six buffy coat and plasma samples, with an input amount of 1 ng for FFPE DNA and gDNA, 0.6 ng for patients’ cfDNA (n = 6) and 0.5 ng for healthy individuals (n = 7) (Supplementary Table S5).

### Statistical analysis

All statistical analyses were performed with RStudio version 3.5.3 (R Foundation for Statistical Computing, Vienna, Austria) which is the graphical user interface of R programming language (R Core Team, 2020). Differences between experimental groups were considered statistically significant when *P* values were < 0.05. Shapiro-Wilk normality test was used to determine whether values follow a normal distribution. F-test was used to assess the equality of variances. Two-sided Mann-Whitney U test was used to compare nuclear cfDNA fragment sizes and levels of control and patient samples. T-Test (student’s t-test or Welch’s t-test) or Mann-Whitney U test was used for comparison of mitochondrial and nuclear copy numbers as indicated. Correlations were analyzed using the parametric Pearson correlation coefficient or the non-parametric rank-based Spearman correlation coefficient as indicated.

### Ethics approval

All procedures performed in this study involving human participants were approved by the ethical standards of the local ethics committee (Ethics Committee of the Rhineland-Palatinate State Medical Association, Mainz, Germany, 11,194) and were in accordance with the 1964 Helsinki declaration and its later amendments or comparable ethical standards. Written informed consent was obtained from all patients.

## Supplementary Information

Supplementary Table S1.

Supplementary Figures.

Supplementary Table S2.

Supplementary Table S3.

Supplementary Table S4.

Supplementary Table S5.

## Abbreviations

cfDNA: Cell-free DNA
cf-mtDNA: Cell-free mitochondrial DNA
cf-nDNA: Cell-free nuclear DNA
CNV: Copy number variation
CRC: Colorectal cancer
ctDNA: Circulating tumor DNA
CTx: Chemotherapy
dPCR: Digital PCR
FFPE: Formalin-fixed paraffin-embedded
mtDNA: Mitochondrial DNA
nDNA: Nuclear DNA
NGS: Next-generation sequencing
SNV: Single-nucleotide variant
VAF: Variant allele frequency
