# Supplementary figures and images for: Comparative analysis of nuclear and mitochondrial DNA from tissue and liquid biopsies of colorectal cancer patients
Source: Sci Rep. 2021 Aug 18;11:16745. doi: 10.1038/s41598-021-95006-6 (PMC8373949; doi:10.1038/s41598-021-95006-6)

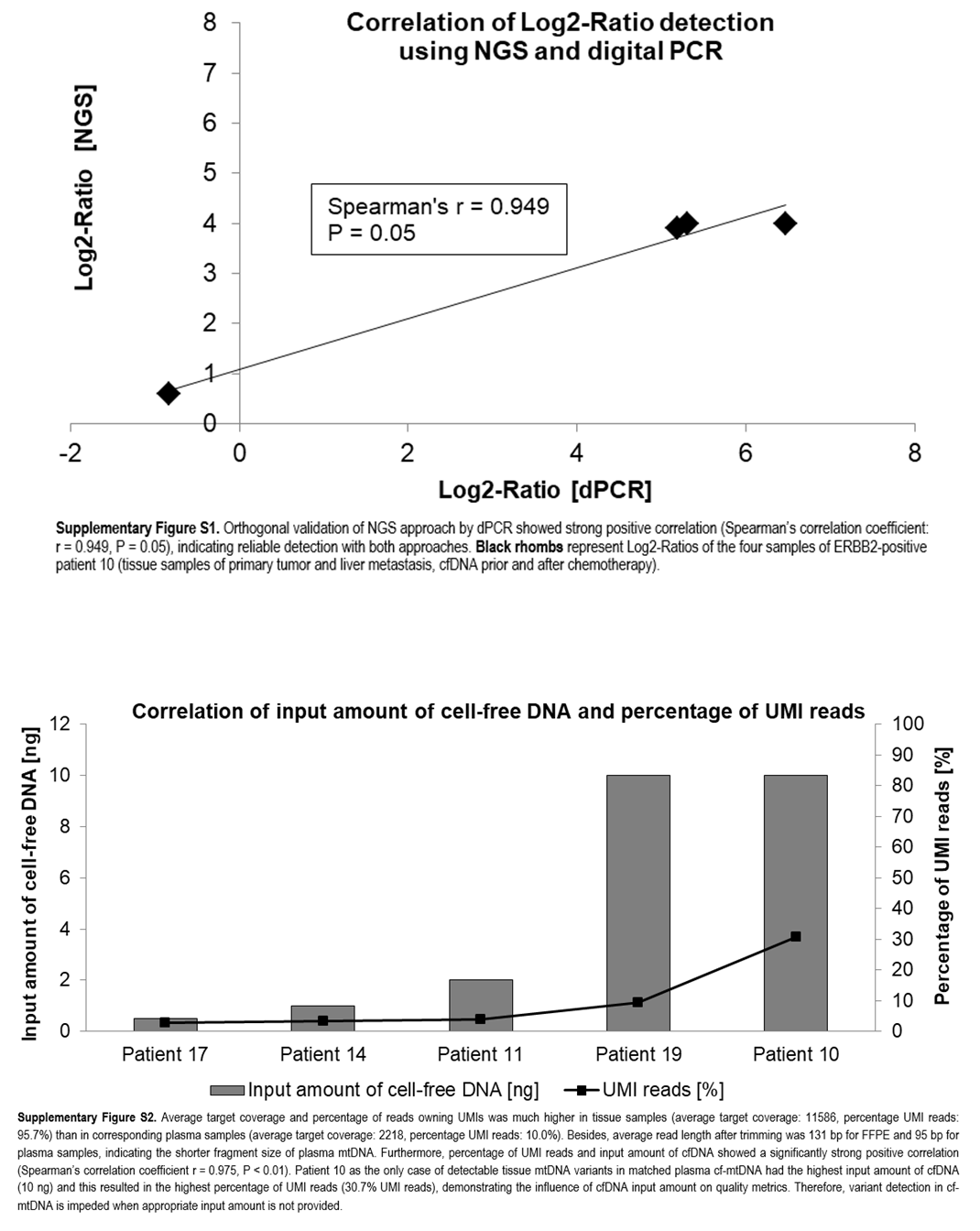

Supplement: Supplementary file 2 — Supplementary Figures. [file 41598_2021_95006_MOESM2_ESM.tiff]
